# Rapamycin Ameliorates Nephropathy despite Elevating Hyperglycemia in a Polygenic Mouse Model of Type 2 Diabetes, NONcNZO10/LtJ

**DOI:** 10.1371/journal.pone.0114324

**Published:** 2014-12-04

**Authors:** Peter C. Reifsnyder, Rosalinda Doty, David E. Harrison

**Affiliations:** The Jackson Laboratory, Bar Harbor, Maine, United States of America; National Center for Scientific Research Demokritos, Greece

## Abstract

While rapamycin treatment has been reported to have a putatively negative effect on glucose homeostasis in mammals, it has not been tested in polygenic models of type 2 diabetes. One such mouse model, NONcNZO10/LtJ, was treated chronically with rapamycin (14 ppm encapsulated in diet) and monitored for the development of diabetes. As expected, rapamycin treatment accelerated the onset and severity of hyperglycemia. However, development of nephropathy was ameliorated, as both glomerulonephritis and IgG deposition in the subendothelial tuft were markedly reduced. Insulin production and secretion appeared to be inhibited, suppressing the developing hyperinsulinemia present in untreated controls. Rapamycin treatment also reduced body weight gain. Thus, rapamycin reduced some of the complications of diabetes despite elevating hyperglycemia. These results suggest that multiple factors must be evaluated when assessing the benefit vs. hazard of rapamycin treatment in patients that have overt, or are at risk for, type 2 diabetes. Testing of rapamycin in combination with insulin sensitizers is warranted, as such compounds may ameliorate the putative negative effects of rapamycin in the type 2 diabetes environment.

## Introduction

Rapamycin (rapa) increases lifespan in mice and several other organisms [1]–[8]. It inhibits mechanistic Target of Rapamycin Complex 1 (mTORC1), which may mediate increased lifespan in caloric restriction studies. Rapa also affects mTORC2 to decrease insulin sensitivity and delay glucose clearance after challenge in B6 mice [9]. This leads to a fear that rapa will cause harm, especially in type II diabetes. The current study directly defines harm and benefit from rapa in a relatively new model of type II diabetes, the NONcNZO10/LtJ. Other studies generally show benefits from chronic treatment with rapa. In a four-strain heterozygous population ([BALB/cJ × C57BL/6J] F1 females × [C3H/HeJ × DBA/2J] F1 males), rapa shows no effect on insulin sensitivity despite delayed glucose clearance and substantially increases lifespans [10]. In a separate heterogeneous mouse population, short-term (2-week) rapa treatment is associated with negative effects on glucose metabolism (poor glucose clearance, insulin insensitivity), while long-term (20-week) treatment is associated with positive effects on glucose metabolism (near-normal glucose clearance and increased insulin sensitivity) as well as reduced adiposity [11]. In KK/H1J mice fed a high-fat diet, rapa impairs glucose tolerance while reducing adiposity [12]. Rapa also reduces adiposity in C57BL/6J mice fed a high-fat diet [13]. Furthermore, rapa treatment in the monogenic obese and diabetic BKS-*Lepr^db/db^* strain suggests that insulin sensitivity is increased by rapa despite continued hyperglycemia. In these studies, rapa also decreases body weight, improves cardiac function, and upregulates markers of mitochondrial biogenesis and fatty acid oxidation in white adipose tissue [14], [15].

In humans, rapa is commonly used as an anti-cancer agent and as an immune suppressant after organ transplant [16]. Hyperglycemia is a side effect of rapa treatment in roughly 10% of patients [16], suggesting that the effect of rapa on glucose homeostasis is dependent upon genetic background and environment. While the prevailing wisdom is that rapa would have a negative effect in patients that have overt, or are at risk for, type 2 diabetes, collectively the data are mixed regarding the benefits vs. hazards of rapa. Therefore, broadening the scope of experimental models may enable researchers to identify conditions that promote either beneficial or detrimental effects of rapa treatment in individuals who are genetically at risk.

The current report describes the chronic effects of rapa treatment in a recently developed polygenic model of type 2 diabetes, NONcNZO10/LtJ (NcZ10). This strain is a recombinant congenic developed in the laboratory of Dr. Ed Leiter at The Jackson Laboratory by backcrossing, on the NON/LtJ background, all of the obesity and diabetogenic QTLs identified in crosses between the NON/LtJ and NZO/HlLtJ strains. The result is a new inbred strain with ∼88% of the genome derived from NON/LtJ and ∼12% from NZO/HlLtJ [17], whose males reliably develop obesity-driven type 2 diabetes.

In contrast to the monogenic BKS-*Lepr^db/db^* strain, which is hyperphagic, morbidly obese (60–80 g), and severely insulin resistant, with juvenile onset of diabetes [18], the NcZ10 males are not hyperphagic, and they develop a moderate obesity (35–45 g) with adult onset diabetes (plasma glucose >250 mg/dL) by 12–20 weeks of age after crossing a body weight threshold of ∼36 g. NcZ10 males develop a moderate hyperinsulinemia with plasma values ∼6 ng/ml [19]. Hyperinsulinemic-euglycemic clamping indicates insulin resistance by 8–10 weeks of age, before onset of hyperglycemia and hyperinsulinemia. Insulin resistance in muscle is associated with reduced Glut-4 [20]. NcZ10 shows elevated triglyceride levels [19] and memory impairment and synaptotoxicity in the hippocampus [21]. Effects of chronic rapa treatment in polygenic type 2 diabetes mouse models that are representative of human disease, such as NcZ10, have not been reported previously. Surprisingly, despite the fact that rapa increases the severity of hyperglycemia in this model, several complications of diabetes — nephropathy, body weight gain, and hyperinsulinemia — were reduced with rapa treatment.

## Materials and Methods

### Animals, Diets, and Caging

Twenty NONcNZO10/LtJ (NcZ10; JAX Stock Number 4456, http://jaxmice.jax.org/strain/004456.html) males were received at 8 weeks of age from The Jackson Laboratory (Bar Harbor, Maine) production barrier facility into the investigator's mouse room at The Jackson Laboratory. Records of pathogens tested are available in health status reports for room D1 at the website: http://jaxmice.jax.org/genetichealth/index.html. The mouse room was maintained on a light/dark cycle of 12 hours, ∼25°C, and 40–50% humidity; all mice were housed with pine shaving bedding and acidified water. The NcZ10 mice were housed in weaning pens (10 mice per pen) and were maintained ad lib on chow diet containing 11% fat (5LA6; PMI, Brentwood, MO, USA) until 12 weeks of age, when 10 mice were switched to 11% fat chow diet (5LA0; PMI) and 10 mice were switched to 5LA0 diet containing 14 ppm (mg of drug per kg of diet) encapsulated rapamycin. It is standard protocol for the NcZ10 model to use at least an 11% fat chow diet in order to drive weight gain over the body weight threshold of ∼36 g necessary to precipitate diabetes onset (see http://jaxmice.jax.org/strain/004456.html). The concentration of rapamycin in the diet was chosen because it had been previously determined to extend lifespan in mice [2]. The encapsulation vehicle, Eudragit S100, is biologically inert and passes through the gut unchanged. It is classified by the FDA as GRAS (generally recognized as safe), and is used in pharmaceuticals to target drugs to the gut.

### Data Collection

All mice were weighed every two weeks. Mice were bled every four weeks by retro-orbital sinus. Mice were fasted for 3–4 hours in a clean cage from 7:00 a.m. to 10–11:00 a.m. before blood collection. This represents a fed blood draw, as mice wouldn't normally feed during this period, but prevents variability in the values due to any morning food ingestion. Plasma glucose (PG) values were measured by glucometer (OneTouch, LifeScan, Inc., USA). The mice had an extra PG measurement taken at 14 weeks of age. Plasma insulin (PI) values were measured at 8, 16, and 24 weeks of age by ELISA (Meso Scale Discovery, Gaithersburg, MD, USA). Spot urine samples were collected over three consecutive days at 25 weeks, and albumin/creatinine ratios (ACRs) were determined using the UniCel DxC 600 Synchron clinical system (Beckman Coulter, Inc., Brea, CA, USA). Two mice from each group were found dead of unknown causes at 26 weeks just prior to sacrifice. Mice were euthanized by CO_2_ at 26 weeks. HbA1c was determined from whole blood at termination using the UniCel DxC 600. Total and HDL cholesterol, glucose, triglycerides, and nonesterified fatty acids (NEFA) were determined from termination serum using the DxC 600. Food consumption was not determined, as the mice produced excessive wastage such that measuring weight of food removed from the grain hopper would have led to a gross over-estimation of food consumption.

### Histology

Pancreas and kidneys were collected at necropsy. Pancreata were fixed in Bouin's and three separate levels were stained with aldehyde fuchsin. Islets were counted from all three levels and scored for size (small, medium, large, and extra large) and degree of granulation (100%, 50–90%, 10–50%, and <10%) by a pathologist. Kidneys were fixed in 10% neutral buffered formalin, and separate sections were stained with H&E, PAS, and anti-IgG. Kidney histology was assessed by a pathologist by scoring 100 glomeruli from each sample for evidence of nephritis and/or hyaline thrombi.

### Ethics Statement

All procedures were approved by the Animal Care and Use Committee of the Jackson Laboratory (Animal Use Summary #99084) to comply with the guidelines of the United States of America Federal government.

### Statistics

ANOVA (JMP, SAS Institute, Inc., Cary, NC) was used for within-strain comparisons for effect of treatment. The nonparametric Wilcoxon rank sum test was used for ACR data due to high variability in the untreated mice.

## Results

### Body Weight and Plasma Glucose

**Figure 1:**
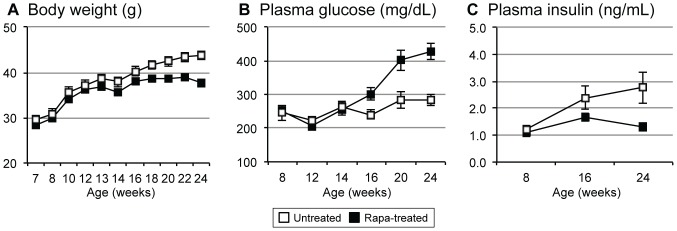
Comparison of NONcNZO10 mice on rapamycin and control diets. Rapa treatment suppresses body weight gain, elevates plasma glucose, and reduces plasma insulin. All 20 mice were fed 11% fat diet until 12 weeks of age, when 10 were switched to an 11% fat diet containing 14 ppm encapsulated rapamycin. (A) Body weight. (B) Plasma glucose. (C) Plasma insulin. Values ± standard error are shown from 8 to 24 weeks of age.

At 12 weeks of age, when half of the NcZ10 were switched to diet containing rapamycin (rapa), the mean ± SE body weight for all 20 mice was 36.7±0.6 g. After the change in diet, those on rapa gained weight in a similar fashion as the untreated mice for four weeks. At 16 weeks, body weight of rapa-treated mice leveled off at 38.5±0.6 g, while untreated mice continued to gain weight until leveling off by 22 weeks at 43.5±1.0 g (Figure 1). Plasma glucose values remained similar between the two groups for two weeks after the diet switch but began to diverge at 16 weeks of age, with values for rapa-treated mice becoming significantly higher. The rapa-treated mice continued to show significantly higher glucose values at 20 and 24 weeks of age even as the untreated mice also transited to glucose values at or above the diabetes threshold of 250 mg/dL (Figure 1). In concordance with the measured PGs, elevated HbA1c values in rapa-treated mice at termination (Table 1) indicated that these mice showed significantly higher average glucose values than untreated over the previous 8 weeks.

**Table 1:** Effects of rapamycin in type 2 diabetes in NcZ10 mice: health and functional test results at end of study (26 weeks of age, 14 weeks of treatment).

Group	*n*	Glucose	HbA1c	TG	Total	HDLD	NEFA	ACR
		(mg/dL)	(% IFCC	(mg/dL)	Chol.	Chol.		(mg/g)
			Units)		(mg/dL)	(mg/dL)		
Control	8	306±31	3.0±0.1	347±27	102±4	86±2	5.4±0.2	151±57
Rapa-treated	8–9	491±15**	5.1±0.3**	333±25	118±5*	97±4*	5.2±0.2	37±16‡

Mean ± SE. Significant difference, control vs. rapamycin-treated within strain, by ANOVA.

**p≤0.0001.

*p≤0.05.

‡p = 0.03 by nonparametric Wilcoxon rank sum used due to high variability in controls.

HbA1c  =  hemoglobin A1c; TG  =  triglycerides; chol.  =  cholesterol; NEFA  =  non-esterified fatty acids; ACR  =  albumin creatinine ratio.

### ACR and the Kidney

**Figure 2:**
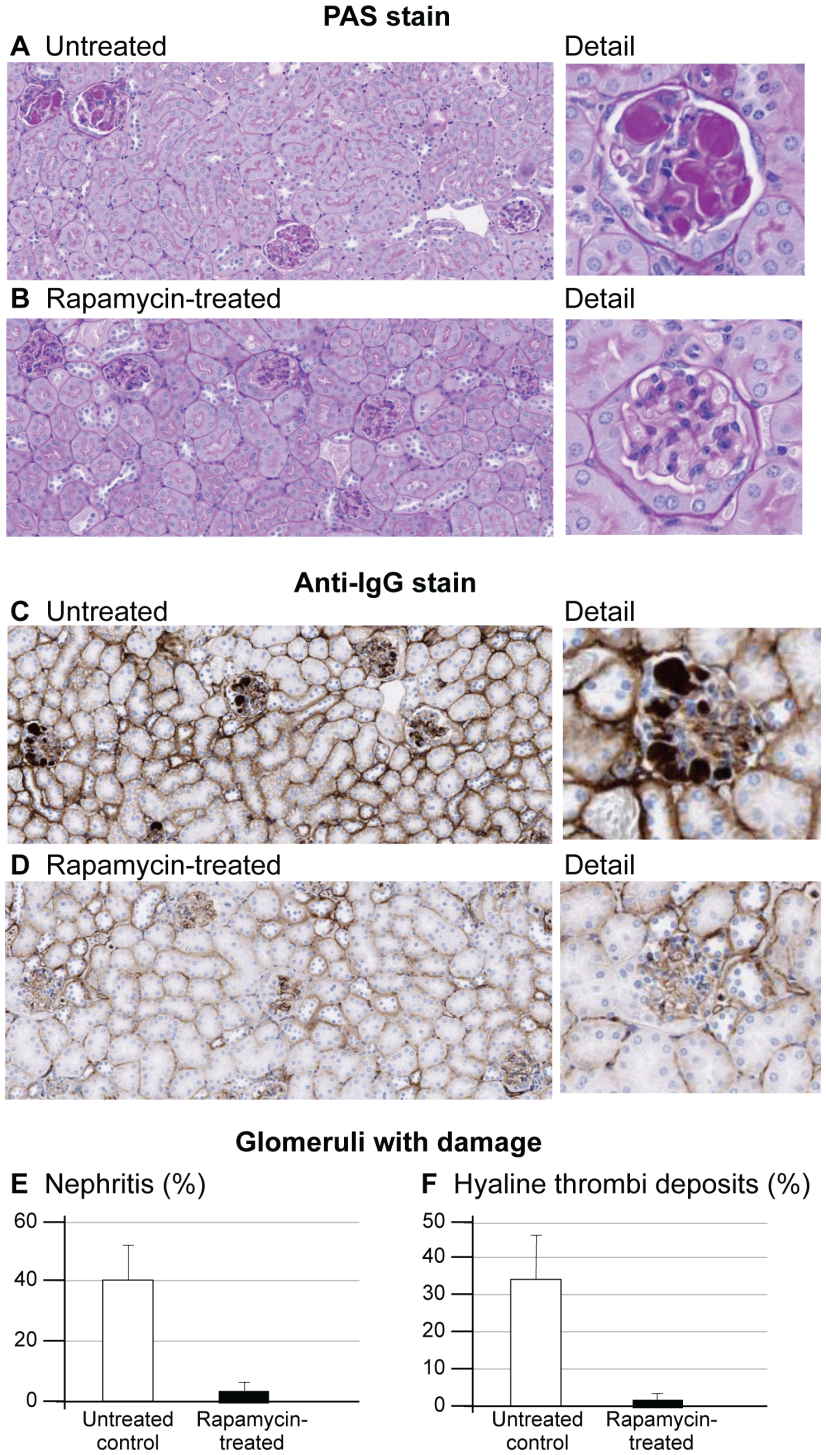
Effect of rapamycin on kidney glomerular morphology in NONcNZO10 mice. Untreated mice (A) show hyaline deposits, likely subendothelial, in the glomerular tuft. These deposits are greatly reduced in rapa-treated mice (B). The deposits are strongly positive for IgG in untreated mice (C), and IgG staining is greatly reduced in rapa-treated mice (D). 100 glomeruli from each mouse were scored for presence of nephritis (E) and/or hyaline thrombi deposits (F), showing quantitatively the reduction in kidney disease by rapa treatment.

Urine albumin/creatinine ratio (ACR) increases with renal damage [22]. ACRs were significantly elevated in the untreated NcZ10 and normal in the rapa-treated mice (Table 1), indicating that rapa protects from renal damage. Foci of inflammation in the kidney were seen in all eight untreated mice assessed for kidney histology but only in 2/8 rapa-treated mice. Also, 6/8 untreated mice showed hyaline deposits in the glomeruli, while only 1/8 rapa-treated mice showed this phenotype (Figure 2A, B). These deposits stained strongly positive for IgG (Figure 2C, D). Scoring 100 glomeruli from each kidney for evidence of nephritis and/or hyaline thrombi (Figure 2E, F) showed that rapa significantly reduced the development of these abnormalities despite exacerbating hyperglycemia.

### Insulin and the Pancreas

**Figure 3:**
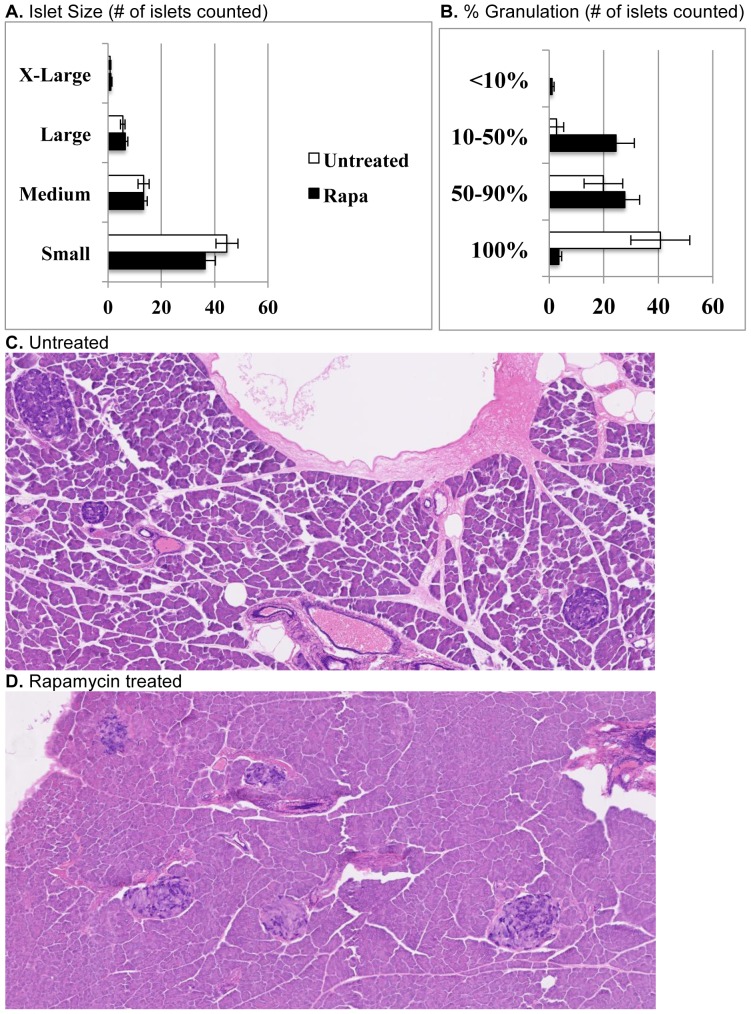
Effect of rapamycin on pancreatic islet morphology in NONcNZO10 mice. (A) Islet numbers and size variability do not differ between untreated and rapa-treated groups. (B, C) Islets in untreated mice are mostly well granulated, but roughly 38% of islets show some degranulation. (B, D) Islets in rapa-treated mice show more extensive degranulation and some fibrous replacement with occasional fibrous encapsulation. Sections were stained with aldehyde fuchsin.

Plasma insulin concentrations showed a mild increase in the untreated NcZ10 over the course of the study (Figure 1). However, the rapa-treated mice showed no increase in plasma insulin, which was significantly lower than in untreated mice at 24 weeks of age (1.3 vs. 2.8 ng/ml, p = 0.02). Histologic examination of the pancreas at three separate levels by staining with aldehyde fuchsin allowed counting and scoring of the islets for size and degree of granulation (insulin storage). While number and size variability of islets did not differ between rapa-treated and untreated mice, a much greater number of islets in the rapa-treated group showed poor beta cell granulation (<50%) and correspondingly fewer islets that were well granulated (Figure 3). Both groups had a similar percentage of islets that show some loss of granulation (50–90% granulated). Islets of treated mice also showed fibrotic foci (Figure 3). Collectively, these observations suggest that the rapa-treated mice were producing, storing, and releasing less insulin, likely explaining the increased hyperglycemia, while the untreated mice showed a pattern consistent with developing insulin resistance. Pancreata in both groups were mildly fatty and had small foci of exocrine inflammation with the exception of two of the untreated mice, which showed necrotic inflammation in the exocrine parenchyma with major fatty replacement.

### Lipids

Total and HDL cholesterol values at termination were significantly higher in the rapa-treated group, while triglyceride and NEFA levels were unaffected (Table 1).

## Discussion

On a broad scale, rapamycin (rapa) increases lifespan [1], [2] and reduces aging rate [3] reliably in genetically heterogeneous as well as inbred mouse populations. Studies in multiple mouse strains have shown that rapa has variable effects on glucose homeostasis and insulin sensitivity [9], [10], [14], [15], and studies in humans have indicated that hyperglycemia is a side effect in roughly 10% of patients [16]. The current report is the first test of rapa in a polygenic mouse model of common human type 2 diabetes, NONcNZO10/LtJ (NcZ10). As expected, rapa accelerated development and increased the severity of hyperglycemia while suppressing hyperinsulinemia. It has been shown that inhibition of mTORC following treatment with rapa or anti-TORC siRNA reduces pancreatic islet mass and proliferation and reduces islet insulin content and secretion [9], [23]–[25]. Histology in the NcZ10 suggests that islet mass and proliferation are not inhibited by rapa treatment, as the number and size variability of the islets were not altered. It is clear, however, that insulin production and secretion were indeed inhibited, as less insulin was being stored in rapa-treated mice and plasma insulin levels were reduced, likely the cause of the more severe hyperglycemia. Because hyperinsulinemia is associated with cancer, cardiovascular disease, and progressive renal disease [26], [27], the prevention of hyperinsulinemia in these mice may have far-ranging benefits despite the exacerbated hyperglycemia. Mice must be aged further to determine if the inhibition of insulin production and the islet fibrosis seen in the rapa-treated mice are benign or if the morphology will become more extensive and pathogenic.

Urine ACR was normalized in the rapa-treated NcZ10; this was likely the result of reduced disease progression in the kidney reflected by reduced general inflammation, glomerulonephritis, and IgG positive hyaline deposits in the glomeruli. The target of rapa, mTORC1, has been implicated in the development of kidney disease. Podocyte-specific activation of TORC induced many features of diabetic nephropathy (DN), and a podocyte-specific knockout of TORC protected hybrid B6/BKS-*db/db* mice from developing DN [28]. Rapa is likely down-regulating an inflammatory response in the kidney as well as inhibiting mTORC1 in the podocyte to reduce nephritis. Since renal disease is associated with elevated plasma insulin values [26], it is also possible that the prevention of hyperinsulinemia may also be a factor in reducing the development of kidney disease. While the difference in pathology between the treated and untreated groups is made clear by urine ACR and histology, at 26 weeks of age disease progression is still relatively mild. Mice will need to be aged longer to drive sufficient disease to understand the altered molecular mechanisms, driven by gene expression, in both the untreated disease-prone mice as well as the disease prevention by rapa-treatment.

Body weight gain was suppressed in the NcZ10. This finding is similar to studies in which obesity was a factor in the treated mice [12]–[15]. Future studies will need to assess fat deposition and gene expression in various adipose tissues in order to unlock the molecular mechanisms of these effects. As blood concentration of rapamycin was not calculated, the fact that the rapa-treated mice showed such a dramatic effect on total body weight lends confidence to the notion that each mouse was receiving a dose sufficient to cause an effect. Had there been a body weight outlier in the rapa-treated group, it would have called into question that mouse's rapamycin intake. NcZ10 is an inbred mouse that is not hyperphagic, so the likelihood of dosage variability is low. Increase in total and HDL cholesterol in NcZ10 is modest and still within the normal range, so the increase may not exert a negative health risk. The non-elevation of triglycerides and free fatty acids with rapa-treatment could be seen as a positive result.

Our study indicates that treatment of a type 2 diabetes mouse strain with rapa, while elevating circulating glucose values, may also reduce certain long-term negative effects associated with hyperglycemia and hyperinsulinemia. It will be important to test the effects of rapa on disease progression over a period of time up to and including lifespan, in NcZ10 and in other type 2 diabetes strains, to determine whether or not the benefits will outweigh the negatives. Studies of rapa combined with other compounds, such as insulin sensitizers, will be useful to determine if the putative negative effects of rapa can be diminished and the positive effects retained. It also will be important to use multiple type 2 diabetes-susceptible strains to determine how this balance of benefits vs. impairments differs among genotypes. Because rapa acts through multiple pathways, such studies may help elucidate the interaction between genes and medicinal compounds and guide translation of results from mouse to human.
