# Correction: Rapamycin Ameliorates Nephropathy despite Elevating Hyperglycemia in a Polygenic Mouse Model of Type 2 Diabetes, NONcNZO10/LtJ

**DOI:** 10.1371/journal.pone.0119748

**Published:** 2015-03-16

**Authors:** The PLOS ONE Staff

Information is missing from the Funding section. The correct funding information is as follows: This work was supported by the National Institutes of Health (AG022308, AG032333, AG038560 to DEH) (http://www.nia.nih.gov/), The Jackson Laboratory’s CA034196. The funders had no role in study design, data collection and analysis, decision to publish, or preparation of the manuscript. This paper is solely the responsibility of the authors and does not necessarily represent the official views of the NIH.
